# Prevention of respiratory tract infections with bacterial lysate OM-85 bronchomunal in children and adults: a state of the art

**DOI:** 10.1186/2049-6958-8-33

**Published:** 2013-05-22

**Authors:** Fernando De Benedetto, Gianfranco Sevieri

**Affiliations:** 1 Pneumology Unit, SS. Annunziata Hospital, Via de’ Vestini, Chieti 66100, Italy; 2 School of Specialization in RespiratoryDiseases, University of Padua, Padua, Italy

**Keywords:** Bacterial lysate, Chronic bronchitis, COPD, Immuno-modulators, OM-85, Respiratory tract infections

## Abstract

Respiratory tract infections (RTIs) are a leading cause of morbidity and also represent a cause of death in some parts of the world. The treatment of RTIs implies a continuous search for stronger therapies and represents an economical burden for health services and society. In this context the prevention of infections is absolutely required.

The use of bacterial lysates as immuno-modulators to boost immunological response is widely debated. Aim of this review is to summarize the main clinical studies on the effect of the bacterial lysate OM-85 in treating RTIs in susceptible subjects - namely children and chronic obstructive pulmonary disease (COPD)-affected adults. Results from clinical trials and recent systematic reviews are reported.

The results show that mean number of RTIs decreases upon treatment with OM-85, as measured by frequency of exacerbations or number of antibiotic courses. Data from systematic reviews indicated that OM-85 is particularly beneficial in children at high risk of RTIs. In COPD-affected adults, clinical studies showed that treatment with OM-85 reduced exacerbations, although systematic reviews did not legitimate the protective effect of OM-85 toward COPD as significant.

The use of OM-85 could be efficacious in reducing exacerbation frequency of RTIs in children and adults at risk. However further high-quality studies are needed to better explain the mechanism of action and confirm the beneficial results of OM85.

## Review

Respiratory tract infections (RTIs) represent a widespread health problem in both adult and paediatric patients and entail elevated economic costs worldwide. To date, two main types of clinical relevant RTIs have been identified: acute exacerbations during chronic bronchitis or Chronic Obstructive Pulmonary Disease (COPD), and recurrent respiratory tract infections (RTIs).

RTIs involve both the upper and the lower respiratory tracts and may be triggered by a wide range of microorganisms. The primitive cause of the disease is generally viral and determined by infections of influenza and parainfluenza viruses, respiratory virus, adenovirus, rhinoviruses [1]. On the other hand, recurrent RTIs (RRTIs) can be caused by different types of bacteria, including *Acinetobacter spp*., *Chlamydia pneumoniae*, *Enterobacteriacee*, *Haemophilusinfluenzae*, *Legionella pneumophila*, *Moraxella catarrhalis*, *Mycoplasma pneumoniae*, *Nocardia asteroids*, *Pasteurella multocida*, *Pseudomonas aeruginosa*, *Staphylococcus aureus*, *Stenotrophomonasmaltophilia*, *Streptococcus pneumoniae* and *Streptococcus pyogenes*.

Strong evidence from epidemiological studies indicates that RTIs are, to date, the major cause of morbidity and mortality in children. According to a 1998 report by the World Health Organization (WHO), acute lower respiratory infections cause 19% of all deaths in children younger than 5 years of age and only in year 2000 they caused 1.9 million deaths among children (95% CI 1.6-2.2 million), 70% of which in Africa and Southeast Asia [2]. Moreover, several studies have pointed out that such infections occur more frequently when linked to predisposition factors such as exposure to tobacco smoke [3], indoor air pollution [4], lack of breast feeding [5], and attending day-care facilities [6].

*Frequency of infections* diminishes *with growth*, yet the average rate of infection in adults remains twice a year. In adults, the most common chronic respiratory condition is COPD [7] in which respiratory viruses and bacterial co-infections are likely to be common [8] and bacterial infections are reported to be associated with 50% of exacerbations [9]. Viral infections that can cause acute bronchitis in healthy subjects are one of the major causes of acute exacerbations in patients with COPD. In the past, the importance of viral infections in the etiology of exacerbations has been largely underestimated and likely contributed to the poor outcome of some antibiotic treatments [8].

One of the traditional therapeutic approaches to prevent and treat RTIs is the administration of antibiotics. However, their efficacy has seriously decreased over the years (likely due to the frequent and improper consumption of antibiotics that has modified susceptibility of the common respiratory pathogens to antibiotics) thus decreasing the benefits for the patient.

In recent years the economical burden of RTIs on the community has been well-established: pharmacological cost involved in the relief of symptoms and in preventing complications, expense due to days lost from work, search for assistance by general practitioner, hospitalization, parental leave from work, other factors, and of course healthcare expenses.

In consideration of current epidemiological and socio-economical data indicating the need for alternative approaches to antibiotic therapies in use today, the aim of this study is to discuss one of the most traditional pharmacological interventions for treating RTIs and COPD, bacterial lysates. Specifically the present review article will focus on the immunomodulator OM-85 and its use throughout the main clinical studies performed in the last years. Results from the main systematic reviews will also be reported.

### Immunomodulation with OM-85 bacteriolysate

One functional and safe approach to prevent and treat acute (ARTIs) and recurrent (RRTIs) respiratory tract infections is increasing non-specifically the immune response or enhancing the organism’s innate defense mechanisms. This can be achieved by using bacterial lysates, which are mixtures of bacterial antigens derived from different inactivated pathogenic microbes. Antigens are obtained by either chemical or mechanical lysis of microrganisms and their lyophilized extract collected from culturing strains of bacteria frequently presents in the respiratory tract.

**Figure 1:**
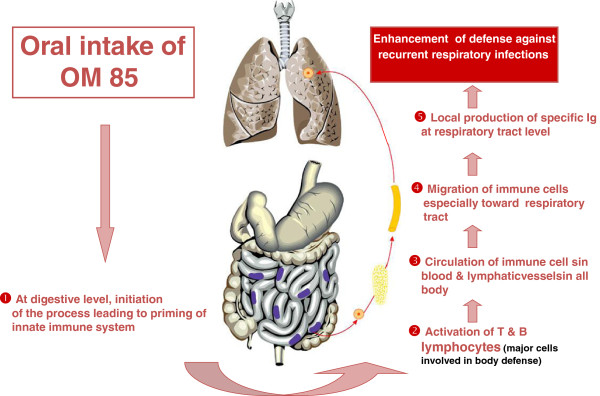
**Five stages undergone by OM-85 from its intake to the generation of antibodies in the respiratory mucosae.** OM-85, as opposite to typical vaccines that are parenterally administered, is administered orally -the paediatric capsule containing 3.5 mg of lyophilzed extract and the adults’ 7 mg. In the intestine lyophilised bacteria reach Peyer’ patches (1). In this reactive lypmphoid tissue, dendritic cells are primed thereby activating lymphocytes and T and B cells, the latter of which will produce antibodies (2). The immune cells are then transported with the lymph into the mesenteric lymph nodes to mature (3). The activated immune cells reach the mucosa of the respiratory tract (4) and stimulate the innate and adaptive immune system as well as the production of secretory IgA antibodies in the respiratory mucosa (5).

OM-85 BronchoMunal® is a bacterial immunostimulant obtained by chemical lysis of G + and G- microorganisms often associated with many respiratory infections, namely *Haemophilusinfluenzae*, *Branhamellacatarrhalis*, *Klebsiellapneumoniae*, *Klebsiellaozaenae*, *Streptococcus* pneumoniae, *Streptococcus pyogenes*, *Streptococcus viridans*, and *Staphylococcus aureus*. The lyophilised extract is administered orally and contains proteins, peptides, and traces of sugar fatty acids, lipoiteichoic acids, and detoxified lipopolysaccharides. The paediatric formulation of OM-85 contains 3.5 mg of the bacterial extract, and the adult’s formulation 7 mg. The main stages undergone by OM-85 at its intake are shown in Figure 1.

Protective effects of bacterial OM-85 are mainly due to its modulatory role in both cellular and humoral responses. In particular, recent findings suggest that the immunoprotective effects of OM-85 are mediated by stimulation of the Th1 cellular response [10] and by inducing Immunoglobulin (Ig) synthesis, mostly IgA, by B cells.

Parallel to its effect on the cellular response, OM-85 also increases the innate immunity in the lungs, by stimulating phagocyte activity thereby increasing destruction of invading pathogens [11].

**Figure 2:**
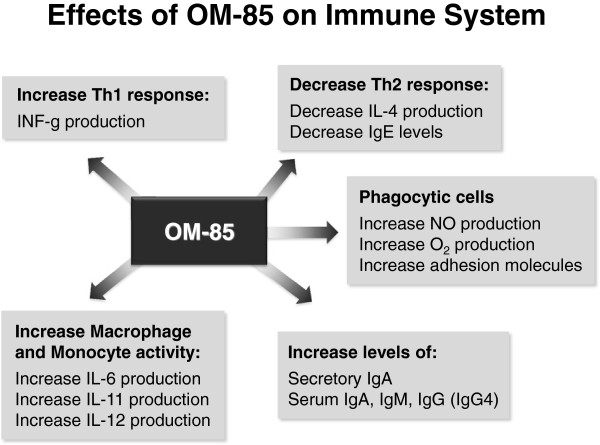
**Mechanism of action of OM-85 [**[11]**].** Ig, Immunoglobulin; INF-γ, Interferon γ; IL, Interleukin; Th1, NO, Nitric Oxide; Type 1 helper T cell; Th2,: Type 2 helper T cell.

Different mechanisms by which OM-85 stimulates phagocytic cells have been proposed. Mauel et al. have shown that bacterial immunomodulators enhance respiratory burst – superoxide and nitrite production- by alveolar macrophages- thereby boosting microbicidal and cytolytic activities [12]. Moreover experimental data have shown that OM-85 increases the expression of adhesion molecules [13] and that a CD-14- independent pathway triggers phagocytes’ activation. The main mechanisms of action of OM-85 is summarized in Figure 2.

The immunostimulant components of OM-85 – namely:porin, murein, and the N-terminal part of lipoprotein [14], probably activate the innate immune systems by binding toll-like receptors (TLRs), and according to a mechanism dependent on TLR signalling adaptor protein MyD88 [10,15,16].

### Evidence of efficacy and safety of OM-85 in children

Increase in both specific and non-specific immunoresponse has been considered a key point when treating RRTIs.

In this regard, the efficacy of OM-85 in boosting immune system and reducing the number of RTIs has been investigated in several clinical trials and analyzed in systematic reviews (Table 1).

**Table 1:** Description of the included clinical trials performed in children

**Study/year**	**Number of patients/treatment**	**Age**	**Population**	**Intervention**	**Follow up**	**Results**
Collet JP et al. 1993 [17]	210 OM-85 BV vs 213 placebo	6-36 mos	Children attending day care centers	1 capsule 10 days/mo for 3 mos	7.5 mos including administration period	No effect at the end of follow-up. During treatment period decrease of risk of RTI (RR 0.52; 95% CI 0.31-0.86)
Jara-Pérez JV et al. 2000 [18]	99 OM-85 BV vs 100 placebo	6-13 yrs	Girls of an orphanage with ARTIs	1 capsule 10 days/mo for 3 mos	6 mos including administration period	Decrease of incidence of ARTIs (p < 0.001); decrease in illness duration, antibiotic consumption, days out of school (p < 0.001).
Gutiérrez-Tarango MD et al. 2001 [19]	26 OM-85 vs 28 placebo	1-12 yrs	RRTIs	1 capsule 10 days/mo; 3 mos. The same six mos later	12 mos including administration period	Decrease of number of ARTIs, antibiotic consumption and duration of ARTI (p < 0.001)
Schaad UB et al. 2002 [20]	120 OM-85 BV vs 100 placebo	26-96 mos	RRTIs	1 capsule/day for 1 mo; 1 capsule/10 days for mos 3, 4 and 5	6 mos including administration period	Lower rate of RRTIs (p < 0.05). largest reduction was in children with ≥ 3RRTIs
Del Rio Navarro BE et al. 2003 [26]	20 OM-85 BV vs 20 placebo	3-6 yrs	ARTIs and low IgG	1 capsule 10 days/mo; 3 mos	6 mos including administration period	Decrease in ARTI occurrence (p < 0.001); decrease in IgG4 level (p < 0.05)
Razi CH et al. 2010 [22]	35 OM-85 BV vs 40 placebo	1-6 yrs	wheezing	1 capsule 10 days/mo; 3 mos	12 mos	Decrease in wheezing attacks (p < 0.001) and of ARTI occurrence (p < 0.001)
Karaca NE et al. 2011 [27]	37 OM-85; 26 no therapy	12-156 mos	IgA deficiency	1 capsule 10 days/mo; 3 mos; no treatment for the following 9 mos. 26 children received one cure-schedule, 11 two or more cure-schedule	50 mos	No difference in RTIs occurrence between the two groups; no development of autoimmune disease

Mo, month; mos, months.

Most of the randomized double-blind placebo controlled clinical trials conducted in children with RTIs have demonstrated the efficacy and the safety of OM-85.

In 1993 Collet et al. studied the effect of treatment with OM-85 on 423 day-care children (6 to 36 months of age) [17]. While the risk for ≥ 4 RTIs at the end of the follow up period was not significantly lower in the active group with respect to the placebo, there was a 48% reduction in the risk of presenting ≥3 episodes of upper respiratory infections in the 3-month treatment period in the treated group.

RTIs are often cause of missed school days in children from 6 to 13 years old: in an elegant study, Jara-Perez et al. have demonstrated the preventive effect of OM-85 on acute RTIs (ARTIs) in 200 girls living in an orphanage, a population of children particularly exposed to microbial contamination and therefore to RRTIs [18]. At the end of the trial, the median number of ARTIs in the active group was 1.0 (0.0-3.0, 5th and 95th percentile) while it was 3.0 (2.0-5.0, 5th and 95th percentile) in the placebo treated group (p < 0.05). Moreover, the Authors showed that treatment with OM-85 significantly reduced the number of missed school days as well as of antibiotic drug courses, and duration of illness (p < 0.001), compared to the placebo treated group.

The efficacy for preventing RTIs in pre-school and school children was confirmed by the study of Gutiérrez-Tarango et al. [19]. In this study, in addition to the decrease in number and duration of RTIs in the active group with respect to the placebo (mean ± SD: 5.04 ± 1.99 vs 8.0 ± 2.55 respectively), the Authors observed a significant reduction in the number of antibiotic courses (p < 0.001 in both cases).

Treatment with OM-85 reduced incidence of ARTIs also in a population of children with recurrent ARTIs aged 3–8 years as reported by Schaad et al. [20]. OM-85 significantly reduced the mean incidence of upper RTIs by 16% in the active group (p < 0.05 with respect to the placebo group). Differences between the two groups increased during the 5-month treatment period and slightly diminished in the follow up month. Reduction in upper RTIs in this study, albeit significant, is less pronounced than that reported by Jara-Perez et al., and Gutierrez et al. The latter trials, however, involved children living in an orphanage [18] and in an area with high pollution level [19]. These settings are both associated with a high susceptibility to RRTIs and would suggest, again, that the efficacy of OM-85 BV is more evident in patients particularly susceptible to RRTIs.

Despite the fact that the increase in exacerbation frequency triggered by infectious agents is an important outcome in children with wheezing and asthma, it usually is included among the exclusion criteria in paediatric clinical trials, and data on this issue are scarce. However, clinicians are seeking for more appropriate treatments, especially since there is accumulating evidence that reversibility of severe atopic diseases decreases over time of onset [21]. Current therapies, including inhaled corticosteroids, have limited efficacy in preventing virus-provoked wheezing attacks in young children. Therefore, primary and secondary preventing strategies are strongly required in those children with RTI-induced wheezing attacks. The efficacy of OM-85 for reducing wheezing attacks was tested by Razi et al. in 75 pre-school children [22]. In this well conducted randomized, double-blind study, the Authors demonstrated a 37.9% reduction in the number of wheezing attacks in treated patients with respect to placebo (p < 0.01), indicating that OM-85 is an effective secondary preventive strategy in children with RTI-induced wheezing attacks both atopic and not. Even though the results of clinical studies on OM-85 to prevent RTIs in asthmatic and atopic children are encouraging, and further well-designed randomized trials in asthma are needed.

Previous studies have shown that OM-85 increases secretory IgA [23], serum IgA [24], serum IgG and serum IgM [25] levels in adults.

However, a small randomized, double-blind placebo-controlled clinical trial performed in children with RRTIs [26] has interestingly shown a significant reduction of IgG4 subclass levels following OM-85 treatment. Since the active role of IgG4 subclass has been demonstrated in type I hypersensitivity reaction, these data seem to infer that the complementary therapy with OM-85 might be useful to reduce RRTIs in children with allergic airway diseases.

The antigenic effect of OM-85 has been tested also in a population of IgA deficient children, with the intent to evaluate whether OM-85 might induce autoimmunity [27]. In this 4-year long prospective study 63 children with IgA deficiency, recurrent febrile infections and RTIs, were recruited and OM-85 was administered to a group of 37 children. At the end of the study the occurrence of RTI did not differ in the two groups of IgA deficient children. However, no clinical and laboratory markers of autoimmunity were observed in the treated and non-treated groups.

The overall effect of OM-85 BV in treating RTIs has been examined also in systematic reviews. In a systematic quantitative review of 13 clinical trials (2,721 patients) testing OM-85, Steurer-Stey et al. found a weak evidence in favour of OM-85 for the prevention of ARTI in children, with a trend for fewer infections [28].

In 2010 Schaad [29] performed a systematic review in order to assess the efficacy of OM-85 for preventing the occurrence of paediatric RTIs. Eight randomized controlled trials were included in the study (children aged 1–12 years) and the Author showed a 26.2% decrease in patients with RRTIs (that is ≥3 RRTI). The results of this study were very heterogeneous probably due to clinical and methodological diversity. The Author indicates that beneficial efficacy of OM-85 was particularly marked in children at high risk of RTIs.

In their recent update of the systematic review “Immunostimulants for preventing respiratory tract infection in children” Del Rio Navarro et al. [30,31] showed that among the 35 trials analyzed (4,060 participants, <18 years, not suffering from asthma, allergy, atopy or chronic respiratory diseases) a 40% reduction of RTIs was in general observed. They hypothesized that the net effect of reducing the incidence of ARTIs is dependent on the background rate of ARTIs and concluded that immunomodulators should be administered only to children with proven high susceptibility to ARTIs [31].

The number of clinical trials analyzed for only OM-85 were 9 (852 participants) with an overall effect of Z = 5.19 (p < 0.001).

Truth is that evaluation of the extent of the effect of OM-85 in reducing the incidence of ARTIs is curbed by discrepancies often due to the high heterogeneity of the studies and to the poor to moderate quality of the trials [32,33].

However, to date several clinical studies have confirmed the efficacy and the safety of OM-85 BV to prevent and treat RTIs in children with infections of different origin.

### Evidence of efficacy and safety of OM-85 RV in adults

The Swiss guidelines indicate the use of immunomodulators among the possible therapeutic options indicated for COPD managementin adults [34]. Moreover, the use of immunostimulants has been demonstrated to be a useful option in COPD management according to the recent recommendation of the Global Initiative of Chronic Obstructive Lung Disease (GOLD), a collaboration between WHO and National Heart Lung and Blood Institute (NHLBI) [35]. However, additional randomized clinical trials are recommended in order to include immunostimulants as regular therapy in COPD patients.

Chronic bronchitis is recognized as a major source of morbidity in the elderly patients who often suffer from recurrent infections of the respiratory tract. The efficacy of OM-85 in preventing RTIs in elderly patients has been demonstrated in several clinical studies as shown in Table 2.

**Table 2:** Description of clinical trials performed in the adult population

**Study/year**	**Number of patients/treatment**	**Age**	**Population**	**Intervention**	**Follow up**	**Results**
Cvoriscec B et al. 1989 [24]	52 OM-85 BV vs 52 placebo	20-69	Chronic brobnchitis	1 capsule/day for 1 mo; 1 capsule/10 days for mos 3, 4 and 5	6 mos including administration period	Decrease of duration of acute episodes and of fever (p<0.001); decrease of antibiotic consumption (p<0.05); increase of T lymphocyte count until 3rd mo after exacerbation (p<0.05); increased serum IgA levels (p<0.05)
Orcel B et al. 1994 [36]	147 OM-85 BV vs 143 placebo	>65	Chronic bronchitis	1 capsule 10 days/mo for 3 mo	6 mos including administration period	28% reduction of lower RTIs entirely due to 40% reduction in the number of episodes of bronchitis (p<0.01)
Collet JP et al. 1997 [37]	191 OM-85 BV vs 190 placebo	OM-85 BV: 65.3±7.7 Placebo: 66.9±7.7	COPD	1 capsule 10 days/mo for 3 mo	6 mos including administration period	No effect in occurrence of acute exacerbation; reduction in number of hospital admissions; decrease in duration of hospitalisation (p=0.037)
Li et al. 2004 [38]	49 OM-85 BV vs 41 placebo	55-82	Chronic bronchitis and COPD	1 capsule 10 days/mo for 3 mo	12 mos including administration period	Decrease in incidence, duration, and severity of acute exacerbation (p<0.01); decrease in consumption of antibiotics
Solér et al. 2007 [39]	142 OM-85 BV vs 131 placebo	22-78	Chronic bronchitis/ mild COPD	1 capsule/day for 1 mo; 1 capsule/10 days for mos 3, 4 and 5	6 mos including administration period	Decrease of acute exacerbations at the end of study (p=0.014). Difference between treatments higher in patients with current or past smoking history
Capetti et al. 2010 [42]	130 OM-85 BV	Not reported	HIV infection- high risk of RTI	1 capsule 10 days/mo for 3 mo	12 mos-comparison performed between year w/o and with treatment	Decrease of antibiotic cycles from 297 to 55. Decrease of hospitalisation from 23 to 6.
Tang et al. 2012 [40]	192 OM-85 BV vs 192 placebo	Not reported	Chronic bronchitis or COPD	1 capsule 10 days/mo for 3 mo	10 wks	Decrease in occurrence of exacerbation (p<0.05); decrease in antibiotic consumption (p<0.05)

In all cases adult dosage was administered.

Orcel et al. evaluated the effect of OM-85, in a randomized controlled clinical trial involving 290 elderly living in an institution [36]. Administration of the bacterial lysate was associated with a 28% decrease in the number of patients with lower RTIs (p < 0.05) which was due to a 40% decrease in the number of episodes of acute bronchitis (p < 0.01). The Authors also observed an overall reduction in antibiotic prescriptions in the treated group compared to the placebo one.

In accordance with the results of Orcel et al., a reduction in the duration of acute episodes of bronchitis and fever was previoulsy observed by Cvoriscec et al. in a randomized clinical study [24]. Also in this case, the treatment with OM-85 decreased significantly the duration of acute episodes of bronchitis (p < 0.001) and consumption of antibiotics (p < 0.05). Interestingly, in addition to the clinical efficacy, the Authors reported a significant increase in the serum IgA levels and in T lymphocyte counts in the OM-85 treated patients until 3 months after the exacerbation, thus confirming the modulatory role of OM-85 in the immune system.

The preventive role of OM-85 in reducing exacerbations in elderly patients with chronic bronchitis and COPD was confirmed in several clinical trials. Collet et al. [37] studied 191 elderly with chronic bronchitis and COPD and reported a 55% decrease in the number of days of hospitalization and in the duration of stay in the active group with respect to placebo (p = 0.037).

In a small later study [38] 90 COPD patients were randomized to receive OM-85 or placebo. At the end of the one-year follow up a significant decrease in incidence, duration and severity of acute exacerbation was observed in the treated patients with respect to the placebo group (p < 0.05 in all cases).

The efficacy of this immunomodulatory agent has been confirmed by Solèr et al. [39] in a slightly younger population with mild COPD or chronic bronchitis. The Authors reported a significantly higher probability for patients treated with OM-85 to remain free of acute exacerbations events (p = 0.014). In particular, the effect of the treatment was more significant among patients suffering from 2 or more acute exacerbation episodes since inclusion in the trial or among smokers (p = 0.001).

In a recent study, 384 patients affected with chronic bronchitis and COPD were involved in a randomized controlled trial comparing OM-85 with placebo on acute exacerbations [40]. At the end of the study the rate of recurrent exacerbations in the active group was significantly decreased (23% with respect to 33% in the placebo group, p < 0.05). Moreover, the results showed also that, among patients with exacerbations, the number of those with recurrent exacerbations was lower in the OM-85 group than in the placebo group (38.7 vs 73.1, p < 0.01), and that the percentage of antibiotics administration was lower in the treated group with respect to the placebo group (37.0% vs 63%, p < 0.05).

The effect of OM-85 was tested also in a group of HIV-positive patients, a patient population for which immunostimulating therapies are actively sought. These patients have a high prevalence of COPD [41] and are at high risk of developing seasonal RTIs. This pilot study encompassed 130 HIV-infected patients at high risk of RTIs [42]. OM-85 was administered for the first year to 65 patients. In view of the satisfactory results obtained in these patients, the treatment was extended to all patients the following year. The number of events in the year before administration and that in the year after administration were compared, and a lower number of antibiotic cycles (which decreased from 297 to 55) and hospitalizations (which decreased from 23 to 6) resulted.

The effect of oral purified bacterial lysates in chronic bronchitis and COPD was analyzed by two recent systematic reviews [28,43]. Steurer-Stey et al. analyzed 13 trials, encompassing 1,971 patients treated with bacterial extracts or placebo [28]. The Authors did not find enough evidence to suggest that treatment could prevent exacerbations. However, treatment with OM-85 BV resulted in improvements of symptoms as assessed by both observers and patients, and shortening of the average duration of exacerbation. In another systemic review Sprenkle et al. evaluated the effect and safety of OM-85 on COPD-affected patients [43]. The Authors examined 13 randomized controlled trials including 2,066 patients and found a non significant trend in favour of OM-85 in reducing frequency of exacerbations even if the safety profile was good. This result might be due to the high heterogeneity of the data and the methodological flaws in some of the trials. Therefore, further larger high-quality randomized controlled trials enrolling subjects with well-defined COPD are necessary to confirm the effectiveness of this agent.

## Conclusions

To date, the efficacy and safety of OM-85 have been demonstrated in several clinical trials, even if systemic analytic reviews underscore the heterogeneity of their study populations. Nonetheless, prevention strategies are fundamental in both children and adult patients affected by respiratory diseases where acute and recurrent RTIs occur. The risk/benefit ratio provided by prevention with OM85 is favorable. Moreover, the encouraging evidence regarding decrease in antibiotic consumption, relief from symptoms and duration of illness represents a logical alternative approach to conventional therapy.

Observations performed in particular patient populations such as those HIV-infected are promising and should encourage to carry out new wide-range investigations. Adopting immunomodulators in the treatment of RTIs in COPD patients could warrant an economical benefit as these agents in COPD patients have proven to decrease the financial burden for both health system and patients [44,45].

Currently, the exact mechanisms of action of OM-85 BV and of its single components are still not well defined. Further studies are required to define these issues, particularly randomized trials enrolling a large population with well-defined diagnosis of respiratory disease could confirm the reported beneficial results observed with treatment with OM-85 and better define the optimal doses, dosing regimens and population target [45,46].

## Consent

Written informed consent was obtained from the patient’s for publication of this report and any accompanying images.

## Competing interests

The authors declare that they have no competing interests.
